# E-cigarette use and intentions to smoke among 10-11-year-old never-smokers in Wales

**DOI:** 10.1136/tobaccocontrol-2014-052011

**Published:** 2014-12-22

**Authors:** Graham F Moore, Hannah J Littlecott, Laurence Moore, Nilufar Ahmed, Jo Holliday

**Affiliations:** 1 Centre for the Development and Evaluation of Complex Interventions for Public Health Improvement (DECIPHer), Cardiff University, School of Social Sciences, Cardiff, UK; 2 MRC/CSO Social & Public Health Sciences Unit, University of Glasgow, UK

**Keywords:** Electronic nicotine delivery devices, Non-cigarette tobacco products, Prevention

## Abstract

**Background:**

E-cigarettes are seen by some as offering harm reduction potential, where used effectively as smoking cessation devices. However, there is emerging international evidence of growing use among young people, amid concerns that this may increase tobacco uptake. Few UK studies examine the prevalence of e-cigarette use in non-smoking children or associations with intentions to smoke.

**Methods:**

A cross-sectional survey of year 6 (10–11-year-old) children in Wales. Approximately 1500 children completed questions on e-cigarette use, parental and peer smoking, and intentions to smoke. Logistic regression analyses among never smoking children, adjusted for school-level clustering, examined associations of smoking norms with e-cigarette use, and of e-cigarette use with intentions to smoke tobacco within the next 2 years.

**Results:**

Approximately 6% of year 6 children, including 5% of never smokers, reported having used an e-cigarette. By comparison to children whose parents neither smoked nor used e-cigarettes, children were most likely to have used an e-cigarette if parents used both tobacco and e-cigarettes (OR=3.40; 95% CI 1.73 to 6.69). Having used an e-cigarette was associated with intentions to smoke (OR=3.21; 95% CI 1.66 to 6.23). While few children reported that they would smoke in 2 years’ time, children who had used an e-cigarette were less likely to report that they definitely would not smoke tobacco in 2 years’ time and were more likely to say that they might.

**Conclusions:**

E-cigarettes represent a new form of childhood experimentation with nicotine. Findings are consistent with a hypothesis that children use e-cigarettes to imitate parental and peer smoking behaviours, and that e-cigarette use is associated with weaker antismoking intentions.

## Background

Arguments regarding the harm reductions that could be achieved, for individual smokers and for public health if tobacco were replaced with e-cigarettes,^1^ have led many public health experts to urge the WHO not to back calls to regulate e-cigarettes as tobacco products or restrict their marketing.^2^ To date, e-cigarette marketing has heavily emphasised smoking cessation benefits.^3^ While such claims have perhaps been made somewhat in advance of robust evidence of effectiveness, a small number of emerging studies do indicate that e-cigarettes may support cessation for some smokers.^4^ ^5^

However, other leading public health experts have argued for greater regulation, pointing to limited evidence regarding direct harms and emerging evidence that e-cigarettes are not adopted primarily for smoking cessation.^6^ Most adult e-cigarette users also smoke tobacco (dual use),^7^ with e-cigarettes being used by some as a means of using nicotine in places where smoking is prohibited.^8^ Internationally, the prevalence of e-cigarette use among adolescents has also increased rapidly in recent years.^9–13^ E-cigarettes do contain some carcinogens and other toxins,^2^ and harm reduction arguments hold little weight were used by young people who would not otherwise have been smoking tobacco.

Public health experts on both sides of debates regarding regulation agree that efforts should be made to prevent young people from taking up e-cigarettes.^2^ ^6^ To date, policy responses to concerns about e-cigarette uptake have led to actions such as plans to ban sales to minors.^14^ More controversially, some have expressed concern regarding visibility of e-cigarettes in places where marketing or use of tobacco has been banned, arguing that this may reverse efforts to denormalise smoking.^15^ Tobacco companies have increasingly invested in e-cigarettes, with some arguing that marketing has targeted youth.^3^ ^16^ ^17^ Hence, while presenting itself as a partner in harm reduction, the industry is arguably seizing new opportunities to introduce young people to nicotine. Governments such as those in parts of the USA have responded to concerns regarding the growing visibility of e-cigarettes by banning their use in public places.^18^ In the UK, the Welsh Government has recently issued a white paper consulting on potential similar legislation.^14^

Arguments relating to potential impacts of the visibility of e-cigarettes centre in part on assumptions that children's perceptions of e-cigarette use as a normative behaviour will increase uptake. Perhaps the most commonly studied source of normative influence on adolescent smoking uptake is parental smoking, with children whose parents smoke more likely to smoke themselves.^19^ While parental influence on e-cigarette use has yet to be investigated, if e-cigarette use is driven by normative factors, children whose parents use e-cigarettes may be more likely to use them. Given that most adult users of e-cigarettes also smoke tobacco, many parents who model e-cigarette use may also model smoking, and their e-cigarette use may be seen by children as a ‘safe’ means of mimicking parental smoking. Peer influences on smoking have also been well established;^20^ although little research has considered whether e-cigarette use represents a means of imitating peer smoking.

Perhaps the most significant concern among those arguing for greater regulation is that childhood e-cigarette use may act as a ‘gateway’ into smoking tobacco.^10^ Opponents of regulating e-cigarettes or limiting their visibility emphasise the lack of evidence for the gateway effect, while expressing concerns that limiting the visibility of safer alternatives may perversely protect tobacco markets.^2^ Indeed, the WHO has described evidence for renormalisation or gateway effects as non-existent. While backing a ban on use of e-cigarettes indoors, the WHO points to uncertainty regarding whether vapour from e-cigarettes is toxic to non-users as a justification for such a move, rather than renormalisation or gateway arguments.^21^ However, the WHO emphasises a need to balance efforts to promote cessation against risks of simultaneously promoting e-cigarettes use among children, also arguing that cessation claims which drive the case against regulation, should be banned from e-cigarette marketing until supported by firmer evidence.^21^

This lack of evidence on both sides of this debate is inevitable. E-cigarettes are a new phenomenon and insufficient time has passed for their harms or benefits to be understood. Experts on both sides have continued to emphasise a lack of evidence for their opponents’ position, while themselves advancing untested hypotheses regarding the harms or benefits of e-cigarettes. Further research is needed to dispassionately support or refute hypotheses being advanced on both sides of the debate.

This paper reports findings from a Wales-wide survey of 10–11-year-old children. First, it examines the prevalence of e-cigarette use, then potential normative influences on children's e-cigarette use, including parental smoking and e-cigarette use and peer smoking. Finally, it tests the hypothesis that never smoking children who report having used an e-cigarette will be more likely to report an intention to take up smoking tobacco; an association which has to date been demonstrated in one study of US middle school children, though has yet to be investigated in younger children or in the UK.^22^

## Methods

### Study design and sample

Child exposure to Environmental Tobacco Smoke (CHETS) Wales 2 was a cross-sectional study of year 6 school children within 75 schools in Wales. Its protocol was reviewed and approved by the Cardiff University Social Science Research Ethics Committee. It replicated the earlier surveys conducted in Wales which examined child's secondhand smoke exposure before and after introduction of smoke-free legislation (CHETS Wales),^23^ and was commissioned and powered primarily to investigate changes in child exposure to smoke in cars. This article reports on questions on e-cigarette use which were included only in the 2014 survey. To ensure that sampled schools were representative of the population of Wales, for CHETS Wales, state-maintained schools with year 6 students were stratified according to high/low (cut-off point identified as average entitlement across whole sample; 17.12%) free school meal entitlement and funding by the Local Education Authority. Within each stratum, schools were selected on a probability proportional to school size. The 75 schools participating in CHETS Wales were invited to take part in CHETS Wales 2; where schools declined, replacement schools were identified from the same stratum. Within each school, one year 6 (age 10–11 years) class was randomly selected by the research team to participate. The samples obtained in CHETS Wales 2 were comparable to CHETS Wales samples in terms of age, sex, socioeconomic status and family structure, indicating that the sampling strategy had been effectively replicated. Consistent with previous analyses from CHETS Wales, no non-response weights were employed.^23^

### Measures

#### Demographics

Children indicated their sex and year and month of birth. To measure socioeconomic status, children completed the Family Affluence Scale (FAS),^24^ comprising measures of bedroom occupancy, car and computer ownership, and family holidays.

#### Parental smoking and e-cigarette use

Children indicated whether any of the following people smoked: (1) father (2) mother (3) stepfather (or mother's partner) and (4) stepmother (or father's partner). Response options were ‘smokes every day’, ‘smokes sometimes’, ‘does not smoke’, ‘I don't know’, ‘I don't have or see this person’. A parent figure was classified as smoking if the child responded ‘smokes every day’ or ‘smokes sometimes’, with all other responses classified as non-smoking parents. Replicating the coding procedures used for parental smoking in CHETS Wales,^23^ children were categorised as having no parent figure who smokes, a father figure (including father or stepfather), mother figure (including mother or stepmother), or a mother and father figures who smokes. The same questions were asked for parental e-cigarette use, with children categorised as having no parent figure who use e-cigarettes, a father figure, mother figure or a mother and father figure who use e-cigarettes. A combined variable was created which indicated whether children had no parent figures who used either tobacco or e-cigarettes, a parent figure who used tobacco only, who used e-cigarettes only or parent figures who used tobacco and e-cigarettes. As only 2% of children reported living with a primary caregiver other than a parent, these variables only included smoking behaviour and e-cigarette usage of parents and step-parents.

#### Peer smoking behaviour

Children were asked to indicate how many of their friends smoked, with response options of ‘most of them’, ‘half of them’, ‘some of them’, ‘none of them’ or ‘I don't know’. Children were classified as having smoking friends if they said that at least some of their friends smoked. No data were available on how many of children's friends used e-cigarettes.

#### Ever smoking and future smoking intentions

Children's ever smoking behaviour was measured by asking ‘Have you ever smoked tobacco? (at least one cigarette, cigar or pipe)’, with response options of ‘yes’ or ‘no’. Future intentions were measured by the question ‘Do you think you will smoke in 2 years’ time?’, with response options of ‘definitely yes’, ‘probably yes’, ‘maybe or maybe not’, ‘probably no’ and ‘definitely no’.

#### Awareness and use of e-cigarettes

Awareness of e-cigarettes was measured by asking children ‘Have you heard of e-cigarettes before this survey?’ Children were asked ‘Have you ever used an e-cigarette?’, with response options of ‘no’, ‘yes, once’ or ‘yes, more than once’. Children were classified as having used an e-cigarette if they responded ‘yes, once’ or ‘yes, more than once’. E-cigarettes were defined as electronic versions of cigarettes which do not give off smoke.

### Consent

Schools signed and returned a commitment form to participate in the study. Parental approval was obtained through letters sent via Royal Mail. In addition to consent forms, information sheets were provided which clearly stated that parents had the option of withdrawing their child from data collection at any time. An ‘opt out’ system was implemented in all but one school. The remaining school requested an ‘opt in’ consent procedure whereby parents/carers informed their child's school if they *did* wish their child to participate in the study. At each data collection session, students were also asked to complete an assent form after having read an information sheet and having had the study explained to them to ensure that they fully understood what they were invited to do, and to give them the opportunity to withdraw from the data collection session if they did not wish to participate.

### Data collection

Data were collected between February and April 2014. Children completed pen and paper surveys, which were placed in sealed envelopes before being collected by researchers. Two researchers attended each data collection to ensure sufficient support and assistance where required. All staff were provided with a data collection protocol and trained by DECIPHer. Teachers were asked to be present, but not to intervene in the data collection in any other way. Briefing sheets were provided for any school staff present, which explained the nature of the study and provided information about the data collection and their anticipated role.

### Statistical analysis

Frequencies and percentages of children who reported using e-cigarettes were calculated for the subsample of children who reported having tried smoking, and for those who reported that they had never tried smoking. Among never-smokers, frequencies and percentages using e-cigarettes were presented by sex, parental smoking behaviour, combined parental cigarette and e-cigarette use, and friends’ smoking behaviour. Binary logistic regression models were used to examine predictors of e-cigarette use. Independent variables were parental cigarette and e-cigarette use (combined into a categorical variable including those who used neither, e-cigarettes and tobacco cigarettes, e-cigarettes only or tobacco cigarettes only), friends’ smoking behaviour, sex and family affluence. Ordinal regression models examined predictors of future smoking intentions, with ORs indicating the relative odds of being assigned to a higher rather than a lower category for the intention to smoke variable (coded from ‘definitely not’=0 to ‘definitely yes’=4). Independent variables were the same as for e-cigarette use, though e-cigarette use was now entered as an independent variable. Owing to the small number of children saying that they might or probably would smoke in 2 years, ordinal models were also conducted with a 3 category dependent variable (combining children who said that they might or would smoke in 2 years), as well as binary models (comparing ‘definitely not’ to all other responses). Comparable results were obtained, hence, we report only models using the 5 category dependent variable. Proportional odds assumption tests for multivariate ordinal models were run using the omodel plug in for Stata V.11, indicating no violations of the proportional odds assumption. To account for the sampling design and non-independence of children within schools, models were adjusted for school-level clustering using the svy commands in Stata V.11.

## Results

### Response rates and sample description

Overall, 114 schools were invited to participate before the target sample of 75 schools was reached (overall response rate=65.8%). Of the 1862 pupils within selected classes, completed questionnaires were obtained from 1601 (86%). In schools where ‘opt out’ consent procedures were followed (n=74 schools, 1810 pupils), 56 children were opted out by parents, 35 children refused and 141 were absent on the day of data collection. Data were obtained from 1578 pupils (87.2%). In the one school which requested opt-in consent, this was given for 23 of 52 children (44.2%), all of whom provided data. Items on e-cigarette use were completed by 1495 children, of whom 51% were female, with a mean (and SD) age of 10.92 (0.40) years. Twenty-one (1.4%) children reported that they had ever smoked tobacco. There were no significant differences between children who did or did not complete questions on e-cigarette use, in terms of age, socioeconomic status (p=0.84) or parental smoking (p=0.50). E-cigarette questions were completed by slightly fewer boys than girls (p<0.01), though overall, an approximately even gender balance was maintained (48.6% boys; 51.4% girls).

### Prevalence of e-cigarette awareness and use

In total, 1014 children (66.8%) reported having heard of e-cigarettes. Among the small number of children who reported having used tobacco (n=21), almost half (47.6%; n=10) also reported having used an e-cigarette. Among never-smokers (N=1467), 77 children (5.3%) reported that they had used an e-cigarette. Table 1 shows frequencies and percentages of e-cigarette use among never smokers by demographic factors, and by parental smoking and e-cigarette use. Overall, 6.5% of male never-smokers and 4.1% of female never-smokers reported having used an e-cigarette.

**Table 1:** Frequencies and percentages of e-cigarette use among children reporting never having smoked a cigarette

		Used e-cigarettes if never smoked Frequency (%)	p Value*
Sex	Boys	46 (6.5)	0.03
	Girls	31 (4.1)	
Parent figures who smoke tobacco†	Neither	30 (3.5)	<0.001
	Mother only	10 (6.6)	
	Father only	10 (5.2)	
	Mother and father figure	25 (11.7)	
Parent figures‡ who use e-cigarettes	Neither	39 (3.5)	<0.001
	Mother only	5 (6.2)	
	Father only	7 (9.2)	
	Mother and father figure	13 (18.6)	
Parent figure smoking and e-cigarette use	Neither	22 (2.9)	<0.001
	Smoke but not e-cigarette	17 (5.2)	
	E-cigarette but not smoke	4 (9.5)	
	Smoke and e-cigarette	21 (11.7)	
Has friends who smoke	Yes	14 (17.7)	<0.001
	No	62 (4.5)	

*p Value from design-adjusted χ^2^ analyses.

†Variable representing whether a parent figure smokes tobacco (regardless of whether they also use e-cigarettes).

‡Variable representing whether a parent figure uses e-cigarettes (regardless of whether they also smoke tobacco).

### Parental smoking, e-cigarette use and dual use

Overall, 231 children (17%) reported that one or more parent figures used e-cigarettes; substantially lower than the percentage (n=615; 39.1%) who reported that at least one parent figure used tobacco. Among never-smoking children who reported that one or more parent figures used e-cigarettes, a large majority (n=168; 72.7%) reported that these parent figure(s) were dual users, who also smoked tobacco. A smaller number (n=20; 8.7%) reported that one parent figure used only e-cigarettes, while the other smoked tobacco. The remaining 18.6% (n=43) reported having only parent figures who exclusively used e-cigarettes. Hence, the vast majority of children who reported that a parent figure used e-cigarettes reported that tobacco was also used by the same parent figure, or in a smaller number of cases, by another parent figure.

### Parental behaviour and child e-cigarette use

As indicated in table 1, for the four category variable representing the number of the child's parent figures who smoked tobacco, the percentage of children reporting having used an e-cigarette increased substantially with parental smoking status. Among never-smoking children who reported that they did not have a parent figure who smoked tobacco, 3.5% reported having used an e-cigarette, whereas for children who reported having a mother and a father figure who smoked tobacco, approximately 1 in 9 (11.7%) reported using an e-cigarette. For parental e-cigarette use, this gradient was steeper. Among children who reported that they did not have a parent figure who used e-cigarettes, 3.5% reported that they had used an e-cigarette, compared to 18.6% of those who reported having a mother and father who used e-cigarettes. Child e-cigarette use was significantly higher among children who reported that parent figures used tobacco and e-cigarettes than among children whose parents did not use e-cigarettes.

### Peer smoking and e-cigarette use

Overall, 97 children (6.2%) reported that at least one friend smoked. Among never-smoking children who reported having friends who smoked, 17.7% reported having tried e-cigarettes as compared to 4.5% of those who reported that they did not have friends who smoke.

### Logistic regression analyses of predictors of e-cigarette use

Table 2 presents ORs and 95% CIs from logistic regression models examining associations of parental smoking, friends’ smoking, sex and family affluence with e-cigarette use. In univariate models, children were more likely to report e-cigarette use if parent figures used e-cigarettes (either solely or in conjunction with smoking). Where parents smoked but did not use e-cigarettes, children were not significantly more likely to have used an e-cigarette. Children who reported having friends who smoked were almost five times as likely to have used an e-cigarette, while boys and children from less affluent families were also more likely to have used an e-cigarette. In multivariate models, however, only parental e-cigarette use (either solely or in conjunction with smoking) and friends’ smoking remained significant predictors of having used an e-cigarette.

**Table 2:** ORs and 95% CIs from logistic regression analyses of e-cigarette use and future smoking intention among 10–11-year-old never-smokers

		E-cigarette use		Future smoking intention		
		Univariate models	Multivariate model (n=1280)	Univariate models	Multivariate model without e-cigarettes (n=1299)	Final model (n=1280)
Parents smoke/use e-cigarettes (reference=neither)	E-cigarettes only	**3.56** (**1.15 to 11.06)**	**3.32** (**1.08 to 10.17)**	1.95 (0.79 to 4.83)	1.32 (0.46 to 3.83)	1.15 (0.39 to 3.63)
	Tobacco only	1.85 (0.93 to 3.69)	1.62 (0.79 to 3.30)	**2.61** (**1.69 to 4.05)**	**2.12** (**1.35 to 3.32)**	**2.09** (**1.30 to 3.44)**
	Both	**4.51** (**2.29 to 8.89)**	**3.40** (**1.73 to 6.69)**	**2.49** (**1.46 to 4.24)**	**2.09** (**1.20 to 3.65)**	**1.88** (**1.07 to 3.31)**
Friends smoking (reference =no)	Yes	**4.53** (**2.37 to 8.65)**	**5.25** (**2.62 to 10.55)**	**5.22** (**3.18 to 8.57)**	**4.05** (**2.27 to 7.22)**	**3.40** (**1.86 to 6.22)**
Sex (reference =boys)	Girls	**0.61** (**0.38 to 0.98)**	0.76 (0.46 to 1.26)	**0.58** (**0.41 to 0.84)**	**0.63** (**0.43 to 0.94)**	**0.66** (**0.44 to 0.97)**
FAS		**0.82** (**0.68 to 0.99)**	0.90 (0.75 to 1.09)	**0.83** (**0.73 to 0.94)**	0.87 (0.75 to 1.00)	0.87 (0.75 to 1.00)
E-cigarette use (reference =no)	Yes	**–**	**–**	**4.28** (**2.52 to 72.8)**		**3.21** (**1.66 to 6.23)**

Significant associations (p<0.05) are highlighted in bold.

FAS, Family Affluence Scale.

### Future smoking intentions

Overall, among never-smokers, almost all children reported that they would definitely not or probably not smoke in 2 years (table 3). Among never-smoking children who reported having used an e-cigarette, few stated that they probably or definitely will smoke in 2 years. However, children who had used an e-cigarette were substantially less likely to report that they *definitely* would not smoke in 2 years, and were more likely to report that they *probably* will not or might smoke in 2 years’ time. Hence, having used an e-cigarette is associated with weaker antismoking intentions.

**Table 3:** Percentage of never-smoking children reporting each level of intention to smoke by whether or not they had used an e-cigarette

	Definitely not	Probably not	Maybe, maybe not	Probably yes	Definitely yes
All never smoking children	1318 (90.3)	105 (7.2)	31 (2.1)	5 (0.3)	1 (0.1)
Children who had not used an e-cigarette	1262 (91.3)	95 (6.9)	22 (1.6)	3 (0.2)	1 (0.1)
Children who had used an e-cigarette	56 (72.7)	10 (13.0)	9 (11.7)	2 (2.6)	0 (0.0)

In univariate models (table 2), antismoking intentions were significantly weaker among children whose parents smoked tobacco (solely or in conjunction with e-cigarettes), among children who reported having friends who smoked, and among boys. Never smoking children, who reported having used e-cigarettes, reported substantially weaker antismoking intentions than those who had not. In a multivariate model including all variables except for e-cigarette use, all significant associations remained, though associations of parental and friends smoking were reduced. The association of e-cigarette use with future smoking intentions remains after adjustment for parental and friends smoking and demographic variables.

## Discussion

Among 10–11-year-old children in Wales, the proportion who had tried an e-cigarette was substantially higher than the proportion who had used tobacco. Hence, data are consistent with concerns, supported by emerging international research, that e-cigarette use appears to represent a new form of early experimentation with nicotine use.^6^ ^9^ ^10^ In addition, consistent with international findings, the vast majority of children who reported that parents used e-cigarettes reported that they were ‘dual users’, who used e-cigarettes as well as tobacco,^7^ indicating that most parents who used e-cigarettes did not completely replace tobacco with them.

Consistent with a body of research showing associations of parental modelling of smoking with uptake of tobacco,^19^ e-cigarette use was also substantially more common among children whose parents used e-cigarettes. However, where parents smoked tobacco though did not use e-cigarettes, children were not significantly more likely to have used e-cigarettes. Hence, there was no evidence that children were using e-cigarettes as a means of mimicking adult smoking in the absence of parental e-cigarette use. It is possible that imitating this behaviour was seen by children as a safer form of experimentation than smoking a cigarette. However, it is also possible that children with parent figures who used e-cigarettes were simply able to access them more easily by, for example, using their parent's e-cigarette, than were the children who did not. While no measure of how many of children's friends used e-cigarettes was included, e-cigarette use was substantially greater among children who reported having at least one friend who smoked.

Before and after adjusting for demographic factors and normative variables, a strong association of e-cigarette use with intention to take up smoking in the next 2 years was observed. This is consistent with a recent US study with older children which found that children who had used an e-cigarette were twice as likely to intend to smoke.^22^ It is important to note that even among children who had used an e-cigarette, few said that they would smoke within the next 2 years. However, substantially fewer children who had used an e-cigarette said that they would definitely not smoke tobacco in 2 years, while a larger proportion said that they might. Hence, children who had used an e-cigarette appeared to have weaker antismoking intentions, indicating greater openness to the possibility of taking up smoking in the near future. Data from the present study are consistent with the hypotheses that children use e-cigarettes to imitate behaviours of parents and peers, and add some tentative support for the hypothesis that use of e-cigarettes may increase children's susceptibility to smoking.

This study is among the first to report on the prevalence and patterning of e-cigarette use in a large survey of primary school-aged children, sampled to be representative of children in the UK, and to the best of our knowledge, the first study to examine associations of e-cigarette use with future smoking intentions in primary school children. However, perhaps the most significant limitation of the study is its reliance on self-report data. While a description of e-cigarettes was given, it might be that some children were unsure what this term meant. There are currently no validated objective means of ascertaining whether or not a child uses e-cigarettes. E-cigarettes are also becoming increasingly differentiated in type, and hence more detailed measures which capture these differences may be useful for future research. The cross-sectional design precludes cause and effect conclusions. For example, while findings are consistent with a hypothesis that e-cigarette use increases children's intention to smoke, intention to smoke may drive e-cigarette use rather than the other way around. Finally, we were only able to demonstrate associations with behavioural intention, which is an imperfect predictor of future behaviour.^25^

It is perhaps premature to be making firm policy recommendations on the basis of an emerging and underdeveloped evidence base; at present, debates on both sides of the current divide are presented with far greater conviction than the evidence base can support. Our findings point to a need to carefully balance harm reduction arguments, which are posed as a justification for limiting regulation of e-cigarettes (and remain contingent on further evidence that e-cigarettes are safe and can be successfully used as a smoking cessation aid), against accumulating evidence of dual use by adults and use among children who would not otherwise be smoking tobacco.

The primary implications of this study relate to a need for further research into children's e-cigarette use. Development of methods to validate children's reports of e-cigarette use and to differentiate between types of electronic nicotine delivery systems is a priority to provide greater confidence in the prevalence estimates obtained from surveys. While we are not able to definitively demonstrate that e-cigarette use leads to uptake of smoking, research adopting longitudinal designs is clearly needed to understand the direction of the associations observed as recognised by the WHO,^21^ as well as to ascertain whether e-cigarette use is followed by subsequent uptake of tobacco. Should future research continue to suggest that childhood e-cigarette use represents an early warning sign that smoking may follow, this may add support for moves toward greater regulation of e-cigarettes, in terms of their advertising and visibility.

While opponents of regulating e-cigarettes appear to be arguing from a default position whereby e-cigarettes are presumed to be associated with few harms unless proven otherwise, the WHO have adopted a position which argues that e-cigarettes should be treated with caution until the potential harms or benefits of e-cigarettes are known.^21^ Research to investigate the safety of e-cigarettes (for users and non-users) and the mechanisms through which they might be offered as effective smoking cessation devices, while limiting children's exposure to them, is necessary in order to better inform public health strategies and reach a compromise between both sides of this debate.

**What this paper adds**

- This study indicates that e-cigarette use represents a new form of childhood experimentation with nicotine which is more common among 10–11-year-olds than tobacco use.
- The majority of children who report that parents use e-cigarettes report that they are ‘dual users’ who also smoke tobacco. Parental ‘dual use’ is associated with children's reported use of e-cigarettes.
- Children who report having used an e-cigarette are less likely to report definite intentions not to smoke and are more likely to report that they might smoke tobacco in 2 years’ time.
